# A case of primary extraskeletal osteosarcoma of the breast

**DOI:** 10.1186/s40792-018-0530-4

**Published:** 2018-09-19

**Authors:** Kanako Kurata, Keisei Anan, Nami Ishikawa, Kenichiro Koga, Michiyo Saimura, Kazuyoshi Nishihara, Toshimitsu Iwashita, Shoshu Mitsuyama, Sadafumi Tamiya, Hideyuki Watanabe, Yutaka Koga, Hidetaka Yamamoto, Yoshinao Oda, Toru Nakano

**Affiliations:** 1 Department of Surgery, Kitakyushu Municipal Medical Center, 2-1-1 Bashaku Kokurakita-ku, Kitakyushu, Fukuoka 802-0077 Japan; 2 Department of Pathology, Kitakyushu Municipal Medical Center, 2-1-1 Bashaku Kokurakita-ku, Kitakyushu, Fukuoka 802-0077 Japan; 3 Department of Radiology, Kitakyushu Municipal Medical Center, 2-1-1 Bashaku Kokurakita-ku, Kitakyushu, Fukuoka 802-0077 Japan; 4 Department of Anatomic Pathology, Graduate School of Medical Sciences, Kyushu University, 3-1-1 Maidashi Higasi-ku, Fukuoka-city, Fukuoka 812-8582 Japan

**Keywords:** Primary sarcoma of the breast, Extraskeletal osteosarcoma, Extraskeletal osteosarcoma of the breast

## Abstract

**Background:**

Primary sarcomas of the breast are rare and account for less than 1% of all primary breast malignancies. We experienced a case of extraskeletal osteosarcoma of the breast that had a unique clinical course and remarkable findings of mammography and magnetic resonance imaging (MRI). A review of the case reports published in the past few decades showed no reports of a case in which a calcified lesion was followed up three different times on mammography, making this a valuable case report.

**Case presentation:**

A 52-year-old woman noticed a right breast mass and underwent a breast examination. Mammography showed a 1.5-cm coarse calcified lesion in the upper outer portion of the right breast. Because fine-needle aspiration (FNA) revealed no suspicion of malignancy, she was followed up. Sixteen months later, the tumor grew progressively to 4.5 cm in size with new calcifications that were fine and irregular in shape and density surrounding an enlarged, coarse calcified lesion. Contrast-enhanced magnetic resonance imaging (MRI) showed a high signal intensity in the periphery of the tumor. Extirpation of the tumor was indicated. The pathological findings were extraskeletal osteosarcoma. She underwent additional resection and latissimus dorsi flap reconstruction at the Department of Orthopedic Surgery.

**Conclusion:**

The present case suggests that mammography findings of a tumor with coarse calcifications that are not typical of benign lesions may be extraskeletal osteosarcoma. A diagnosis must be made as early as possible in order to improve the prognosis of this disease.

## Background

Primary sarcomas of the breast account for < 1% of all primary breast malignancies [1]. Among those sarcomas, extraskeletal osteosarcoma is extremely rare. Generally, the tumor presents as a progressively enlarging soft tissue mass. The prognosis for patients with this disease is poor, and most patients succumb to the metastatic disease within 2 to 3 years after the initial diagnosis [2].

In this case report, we describe the unique clinical course of the disease and the remarkable findings of mammography and magnetic resonance imaging (MRI).

## Case presentation

**Fig. 1:**
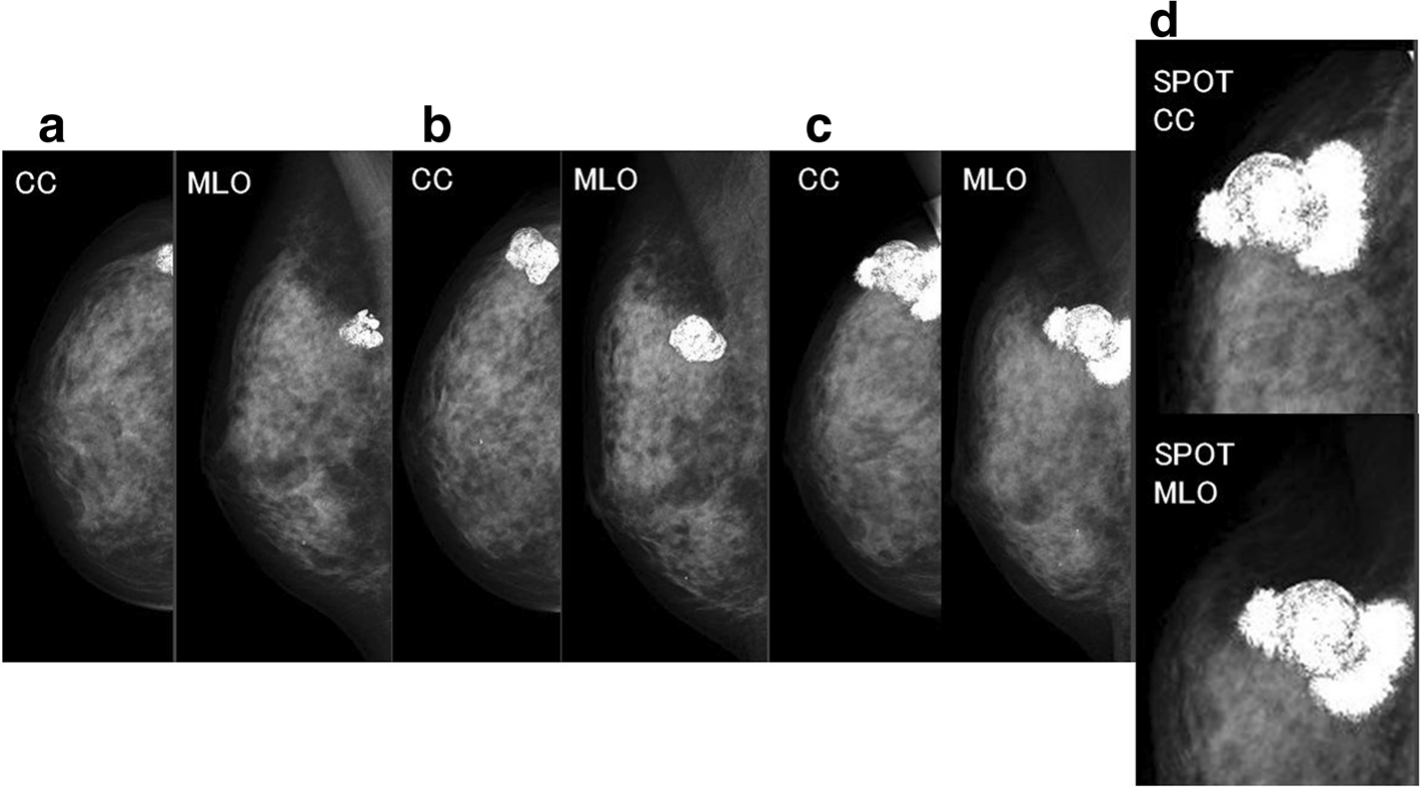
Mammography. **a** At the initial visit, it revealed a 1.5-cm coarse heterogeneously high-density calcified lesion at the upper outer portion of the right breast. **b** About 8 months after our first medical examination, it revealed a slight enlargement of the coarse calcification. **c**, **d** About 16 months after our first medical examination, the lesion had grown to 4.5 cm, and new microcalcifications that were fine and irregular in shape and density appeared around the enlarged original coarse calcified tumor

**Fig. 2:**
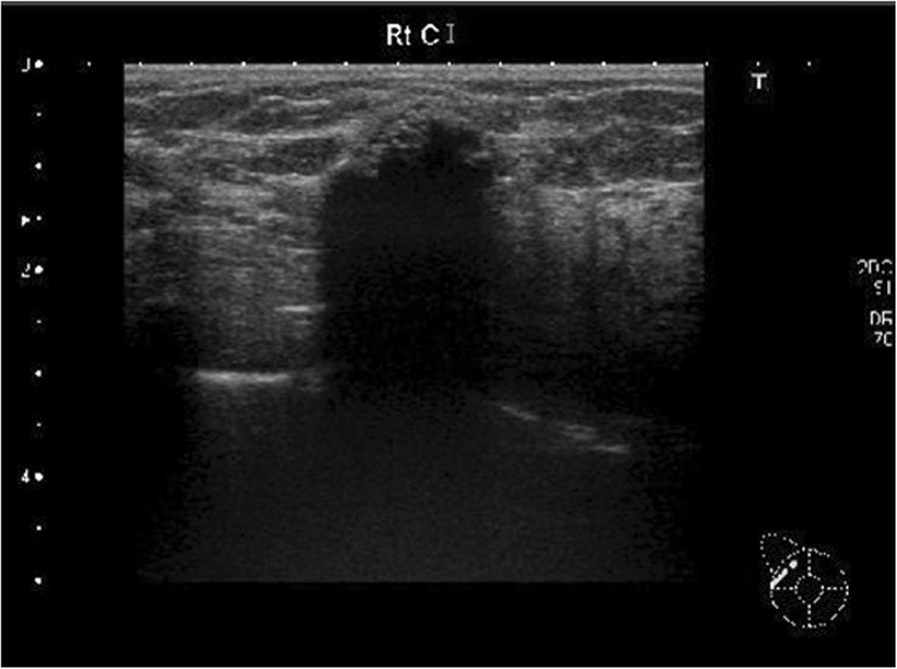
Ultrasonography. A 1.7-cm-diameter relatively smoothly marginated hypoechoic mass containing multiple calcifications

A 52-year-old woman noticed a right breast mass and underwent a breast examination at a local breast clinic, after which she was referred to our hospital for a further investigation. The patient’s medical history included early gastric cancer and early esophageal cancer, and her family history included breast cancer in a younger sister and gastric cancer in a grandfather. Mammography revealed a 1.5-cm coarse heterogeneously high-density calcified lesion at the upper outer portion of the right breast (Fig. 1a). Ultrasonography (US) showed a hypoechoic mass containing multiple calcifications with relatively smooth borders. The internal characteristics were unclear due to the calcifications (Fig. 2). Fine-needle aspiration cytology of the tumor showed small clusters of either normal or benign epithelial cells without any marked atypia. Since no malignancy was noted, we did not perform a core needle biopsy, and the patient was subsequently followed up.

**Fig. 3:**
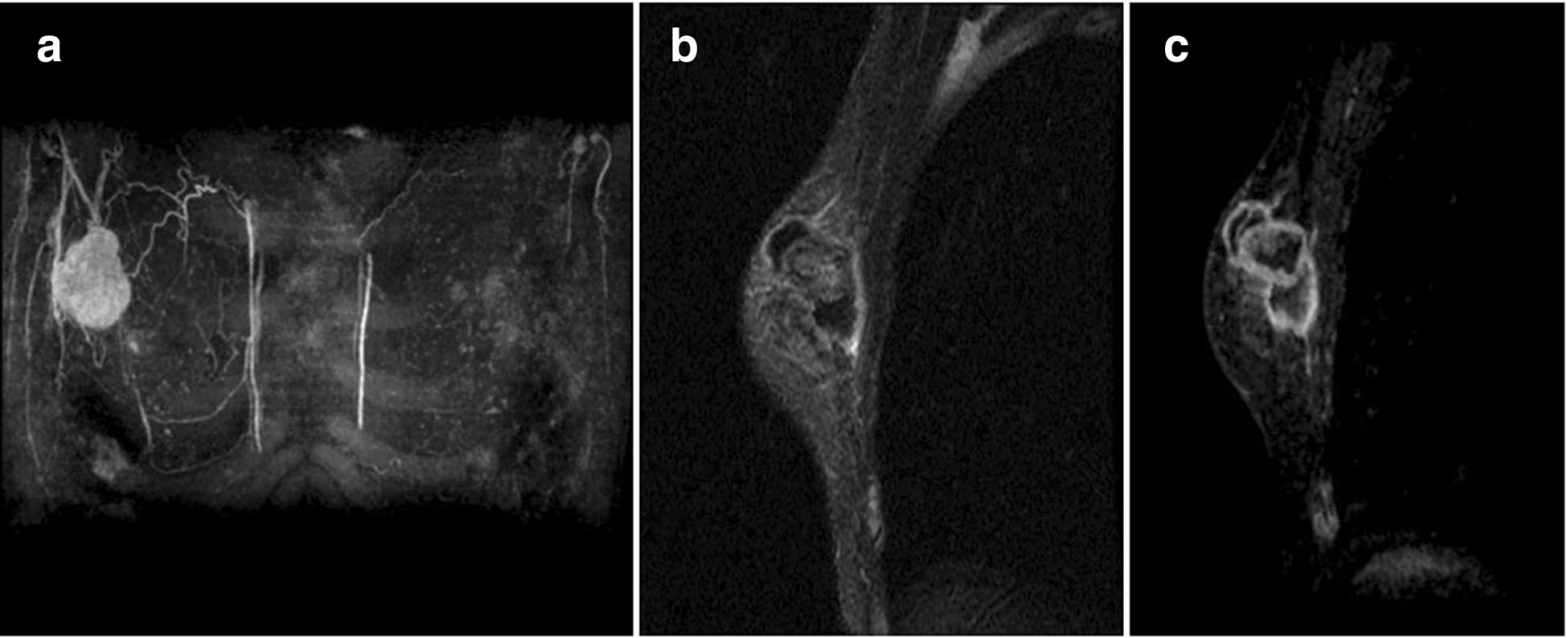
Magnetic resonance imaging. **a** A 4.5-cm-diameter mass at the upper outer portion of the right breast. **b** Fat suppression (FS) T2-weighted imaging (WI): A high signal intensity at the periphery and center of the tumor. **c** Gadolinium (Gd) FS T1WI: A high signal intensity at the periphery of the tumor but a low signal intensity in the central area

**Fig. 4:**
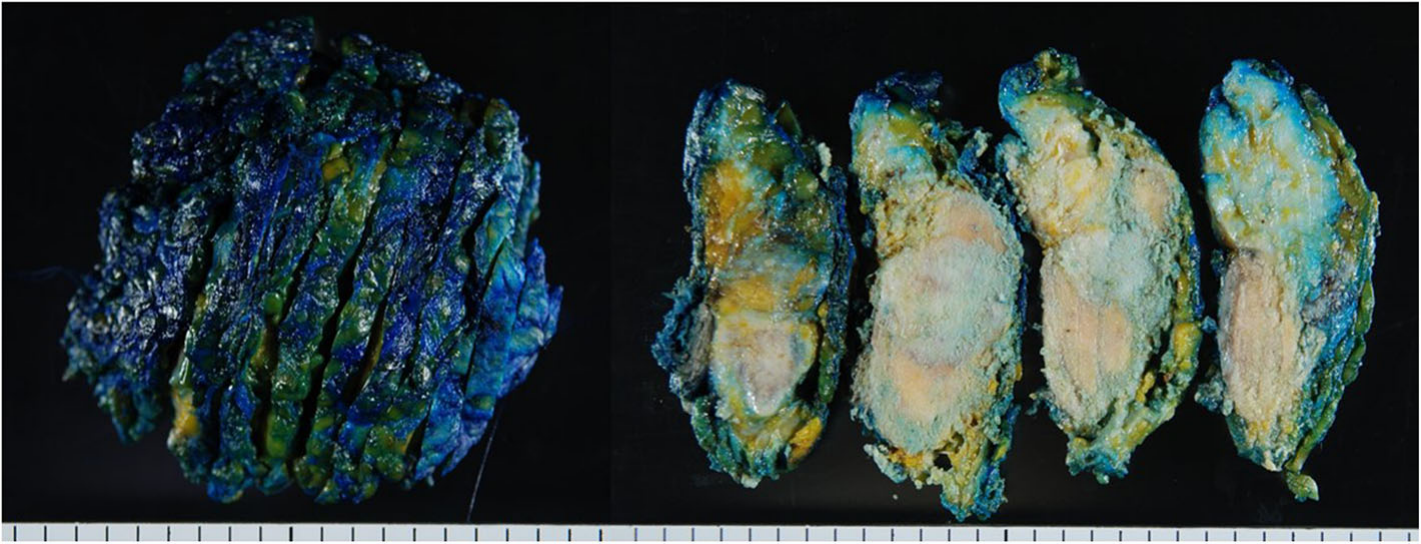
Excised specimen. The center of the tumor is whitish, and both lateral portions are yellowish

**Fig. 5:**
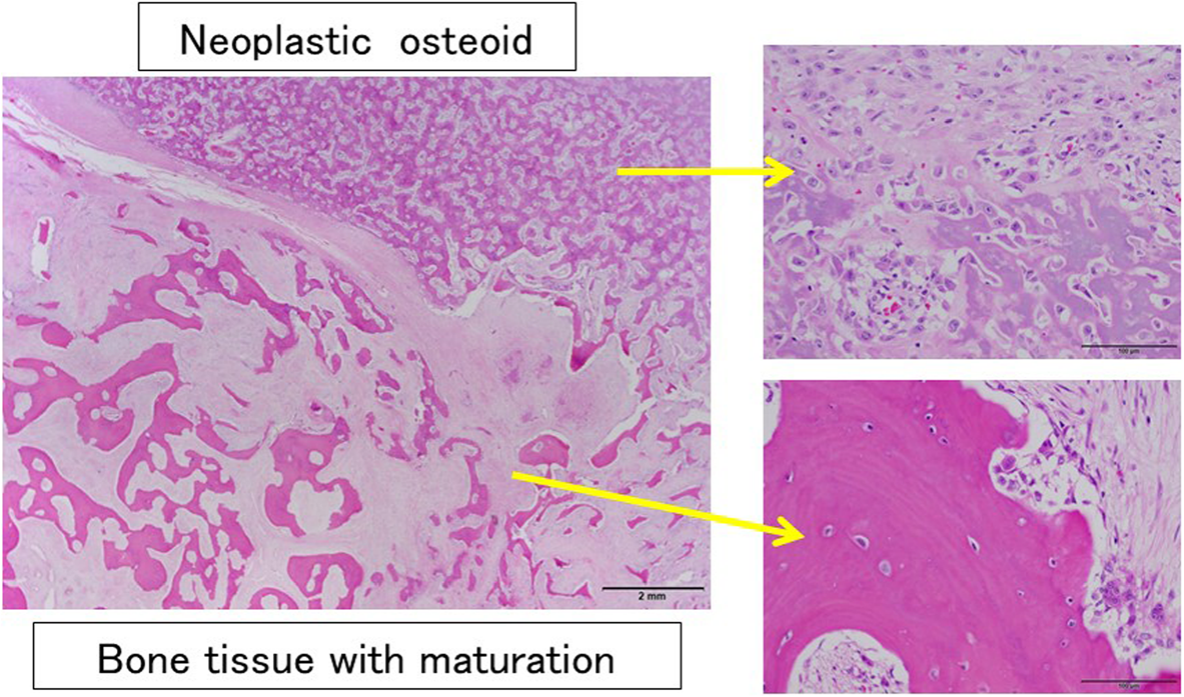
The histopathological examination. The proliferation of atypical cells with oval to round nuclei and eosinophilic cytoplasm with abundant osteoid and bone formation are seen at the periphery of the tumor. The center of the tumor is composed of hypocellular fibrous maturation. Mitotic figures are frequently seen, but abnormal mitotic figures are not evident

**Fig. 6:**
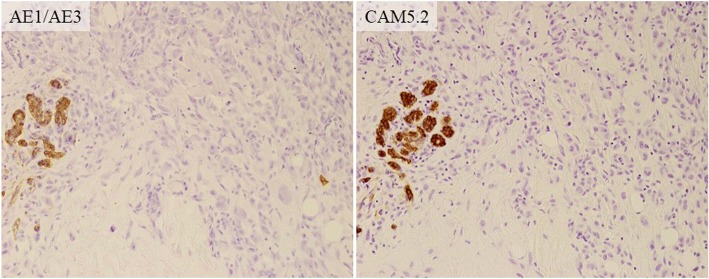
Immunohistochemistry. (× 200) The atypical cells were negative for AE1/AE3 and CAM5.2, while the residual normal breast tissue on the left side was positive for these factors

About 8 months after our first medical examination, mammography revealed a slight enlargement of the coarse calcification (Fig. 1b), but US showed the same size tumor. About 13 months after our first medical examination, the mass was 2.3 cm in size, and after another 3 months, it had grown to 4.5 cm, and new coarse calcifications that were irregular in shape and different in density from the initial one appeared around the enlarged original coarse calcified tumor on mammography (Fig. 1c, d). US showed a mass that was covered by coarse calcifications which had remarkably increased in size. MRI revealed a 4.5-cm mass at the upper outer portion of the right breast. Fat suppression (FS)-T2-weighted imaging (T2WI) showed a high signal intensity at the periphery and center of the tumor. Gadolinium (Gd)-enhanced FS-T1WI showed a high signal intensity at the periphery of the tumor but a low signal intensity in the central area (Fig. 3a–c). A core needle biopsy showed a nodule with bone formation (ossification) that was not malignant. The serum alkaline phosphatase level was 292 U/L (106–322 U/L) at the initial visit but had increased to 548 U/L when the tumor grew. Other hematological findings, including lactate dehydrogenase levels, were within the normal ranges. Extirpation of the tumor was performed. Because the tumor had infiltrated the greater pectoral muscle, we resected a portion of it (Fig. 4). The pathological findings were extraskeletal osteosarcoma. Sectioning showed the proliferation of atypical cells with oval to round nuclei and eosinophilic cytoplasm with abundant osteoid and bone formation in the periphery of the tumor. The center of the tumor was composed of hypocellular fibrous maturation, and mitotic figures were frequently seen, but abnormal mitotic figures were not evident (Fig. 5). Immunohistochemically, the atypical cells were positive for MDM2, CDK4, and p16 but negative for AE1/AE3, CAM5.2, and p53 (Fig. 6). There were no histopathological findings of breast cancer, malignant phyllodes tumor, carcinosarcoma, or dedifferentiated liposarcoma.

After the pathological diagnosis of primary extraskeletal osteosarcoma in the breast was made, the patient received doxorubicin and ifosfamide (AI, three cycles) as adjuvant chemotherapy and then underwent additional resection with latissimus dorsi flap reconstruction at the Department of Orthopedic Surgery. She then resumed AI (two cycles), but at 16 months after the first extirpation procedure, multiple lung metastases appeared, and her treatment was changed to doxorubicin and cisplatin (AP, two cycles). The lung metastatic lesions grew, and the treatment was changed again to trabectedin (seven cycles); however, it had no effect. She is currently receiving eribulin and radiotherapy for multiple lung metastases.

### Discussion

Primary sarcomas of the breast are rare and account for < 1% of all primary breast malignancies [1]. Among those, extraskeletal osteosarcoma is extremely rare. Extraskeletal osteosarcoma is a malignant mesenchymal neoplasm that produces osteoid, bone, or chondroid material and is located in the soft tissue without attachment to the skeleton [2]. Although osteosarcomas of the bone occur mostly during the first two decades of life, extraskeletal osteosarcomas are rarely encountered in patients under 40 years of age [2, 3]. Some studies have reported that a tumor is likely to be caused by a history of trauma to the breast or radiation therapy for a malignant neoplasm [4], preceding fibroadenoma or phyllodes tumor [1]. The most common presentation is a progressively enlarging mass. In our case, the patient had no medical history of breast or preceding fibroadenoma or phyllodes tumor, but a coarse calcified lesion was observed in her breast. The tumor started to grow progressively in the course of follow-up, similar to other cases reported in the literature.

In the present case, changes in the coarse calcified lesion on mammography were remarkable, and the contrast-enhanced MRI findings were characteristic. When the tumor grew, new microcalcifications that were fine and irregular in shape and density appeared on mammography. The characteristics of the calcified lesions differed substantially from those noted at the initial visit. Because the newly appeared microcalcifications surrounded the initial coarse calcified lesion, the new lesion may have originated from a component of the initial one. Other reports have described mammography findings such as “an ill-defined dense mass containing soft calcifications” [5] or “an oval, fairly dense, and well-delineated mass” [6]. In the present case, the mammography findings both at the initial visit and at the new lesion appearance were clearly coarse calcifications. There might be no characteristic findings on mammography for primary osteosarcoma of the breast. This case report was considered to be valuable due to the fact that the calcified lesions had been followed up over a long period of time by mammography.

Extraskeletal osteosarcoma has a very poor prognosis, and approximately 75% of patients die of their disease within 5 years of the diagnosis [7]. A study reported that the 5-year overall survival probability of primary osteogenic sarcoma of the breast was 38%, and the 10-year estimated survival was 32% [1]. These data are consistent with the observation that metastases developed in patients within 3 years of the diagnosis, and those patients died of progressive disease within a relatively short time thereafter [1]. Treatment should include complete surgical removal of the tumor with an adequate margin [1, 8, 9]. The local recurrence rate was 67% for patients treated with local excision (a biopsy or tylectomy) and 11% for those treated with mastectomy [1]. As adjuvant chemotherapy, methotrexate and bleomycin, cyclophosphamide, CDDP, doxorubicin, and ifosfamide have been shown to be effective, as well as a therapy for osteosarcoma of the bone [6, 10, 11]. However, no standard regimen has been established for extraskeletal osteosarcoma. In the present case, because the resection margin was close, additional resection and chemotherapy were performed. A diagnosis must be made as early as possible in order to improve the survival of this disease.

## Conclusions

If a tumor with coarse calcifications that are not typical of benign lesions is noted on mammography, it could be extraskeletal osteosarcoma, as in the present case. Contrast-enhanced MRI may be useful for ruling out malignant neoplasms. Further investigations regarding the mechanism of tumor development, imaging findings for early detection, and the optimum treatment are imperative for addressing particularly high-grade neoplasms.

## Abbreviations

FNA: Fine-needle aspiration
FS-T1WI: Fat suppression -T1-weighted imaging
FS-T2WI: Fat suppression -T2-weighted imaging
MRI: Magnetic resonance imaging
US: Ultrasonography
